# Management of ruptured pulmonary hydatid cyst presenting with massive haemoptysis in a 4-year-old child: a case report

**DOI:** 10.1093/jscr/rjag350

**Published:** 2026-05-06

**Authors:** Reem Amro, Ghassan Abu-Sharikh, Kenana Altell, Mohsen Alqawasma, Lana Abuzahra

**Affiliations:** Department of Pediatrics, Alia Hospital, Hebron, Palestine; Department of Emergency Medicine, Alia Hospital, Hebron, Palestine; Department of Medicine, Hebron University, Hebron, Palestine; Department of Medicine, Hebron University, Hebron, Palestine; Department of Medicine, Palestine Polytechnic University, Hebron, Palestine

**Keywords:** ruptured pulmonary hydatid cyst, pediatric, rare presentation

## Abstract

Hydatid disease (HD) is a zoonotic parasitic infection caused by *Echinococcus granulosus* and remains endemic in many regions. In children, pulmonary involvement is more common than hepatic disease and is often asymptomatic or mildly symptomatic. Massive hemoptysis as an initial presentation in early childhood is extremely rare. We report a case of a previously healthy 4-year-old boy who presented with a 3-day history of productive cough and massive hemoptysis without fever or systemic symptoms. Chest radiography showed right-sided consolidation, and contrast-enhanced computed tomography revealed a large cavitary lesion in the right middle lobe suggestive of a ruptured hydatid cyst. The patient underwent successful video-assisted thoracoscopic surgery-assisted mini-thoracotomy excision with chest tube placement, followed by albendazole therapy. Recovery was uneventful with excellent outcome. This case highlights the need to consider pulmonary HD in the differential diagnosis of hemoptysis in children living in endemic areas and supports minimally invasive surgical management when feasible.

## Introduction

Hydatid disease (HD) is a widely distributed zoonotic infection that remains endemic in several regions worldwide, including the Mediterranean basin, Central Asia (particularly the Tibetan plateau), Australia, South America, and Northern and Eastern Africa [1]; it is a parasitic infection caused by *Echinococcus granulosus* for which human have no role in its life cycle, but get infected accidently [2].

The presentation of HD varies by age. In pediatric population pulmonary HD is prominent, unlike adults, where hepatic involvement is common [3]. The location also plays a major role in determining the size and the growth rate of HD. Tissue elasticity probably has a major role in limiting the growth rate. Compare to dense organs, soft organs growth is faster. It is reported that lung cysts grow at a higher rate than liver cysts. Negative intrathoracic pressure and high elasticity may result in rapid growth of a pulmonary cyst, whereas the compact tissue and hepatobiliary system in the liver probably limit HD growth [4].

Infected patients may remain asymptomatic for months to years. When the lungs are infected manifestations like cough, dyspnea, fever, nausea, vomiting, and thoracic deformations start to appear. Children and adolescents may remain asymptomatic even with large lesions, due to high elasticity of their lung parenchyma [5]. Pulmonary HD presenting with massive hemoptysis in early childhood is exceedingly rare, as most pediatric cases present with mild respiratory symptoms or remain asymptomatic, even in the presence of large cysts.

Herein, we report a rare pediatric case of pulmonary HD in a 4-year-old boy who presented with episodic productive cough and massive hemoptysis (approximately one cup in volume), without other associated symptoms. Contrast-enhanced chest computed tomography (CT) revealed a cavitary lesion involving the anterior segment of the right upper lobe. The patient underwent right-sided video-assisted thoracoscopic surgery (VATS) assisted mini-thoracotomy for hydatid cyst excision, which was completed successfully without complications.

## Case report

A 4-year-old male child, previously healthy with no significant past medical or surgical history and no known drug allergies, presented with a three-day history of episodic productive cough associated with massive hemoptysis, characterized by frothy, blood-tinged sputum of approximately one cup per episode, without fever, respiratory distress, weight loss, sweating, chest trauma, or other systemic symptoms. His blood pressure was 90/50 mmHg. Initial evaluation included complete blood count, liver and renal function tests, and chest imaging. Laboratory investigations revealed eosinophilia and basophilia, consistent with a parasitic infection, with detailed results presented in Table 1.

**Table 1:** Laboratory results.

Test	Result	Unit	Reference range
White blood cells (WBC)	12.7	cell/L	5.5–15.5
Neutrophils %	83.6	%	37–92
Lymphocytes %	8.5	%	
Monocytes %	8.4	%	3–7
Eosinophils %	**4.1**	%	1–3
Basophils %	**1**	%	0–0.75
Neutrophils (absolute)	7.2	K/μl	1.5–8
Lymphocytes (absolute)	1.8	K/μl	0.7–4.8
Monocytes (absolute)	0.9	K/μl	0.3–1
Eosinophils (absolute)	0.3	K/μl	0–0.8
Red blood cells (RBC)	4.58	M/μl	3.9–5.3
Hemoglobin (Hb)	**10**	g/dl	10.5–14
Hematocrit (HCT)	**31.8**	%	33–42
Mean corpuscular volume (MCV)	**69.3**	fl	70–74
Mean corpuscular hemoglobin (MCH)	**21.8**	pg	27–31.2
Mean corpuscular Hb concentration (MCHC)	32.3	g/dl	31–35
Red cell distribution width (RDW)	14.1	%	11.5–14.5
Platelet count	193	×10^3^/μl	150–450
Mean platelet volume (MPV)	8.4	fl	7–11
Platelet distribution width (PDW)	20.4	—	—
Erythrocyte sedimentation rate (ESR)	**35 ↑**	mm/hr	0–10
C-reactive protein (CRP)	**54.6**	g/L	0–5
Prothrombin time (PT)	16	s	11–15
INR	**1.23**	-	-
Activated partial thromboplastin time (aPTT)	**33**	s	25–40
Creatinine	0.35	mg/dl	0.7–1.2
Blood urea nitrogen (BUN)	14	mg/dl	7–50
Aspartate aminotransferase (AST/GOT)	21	U/L	0–41
Alanine Aminotransferase (ALT/GPT)	10	U/L	0–41
Sodium (Na)	134	mmol/L	135–145
Potassium (K)	4.0	mmol/L	3.5–5.3
Chloride (Cl)	104	mmol/L	98–110

**Figure 1:**
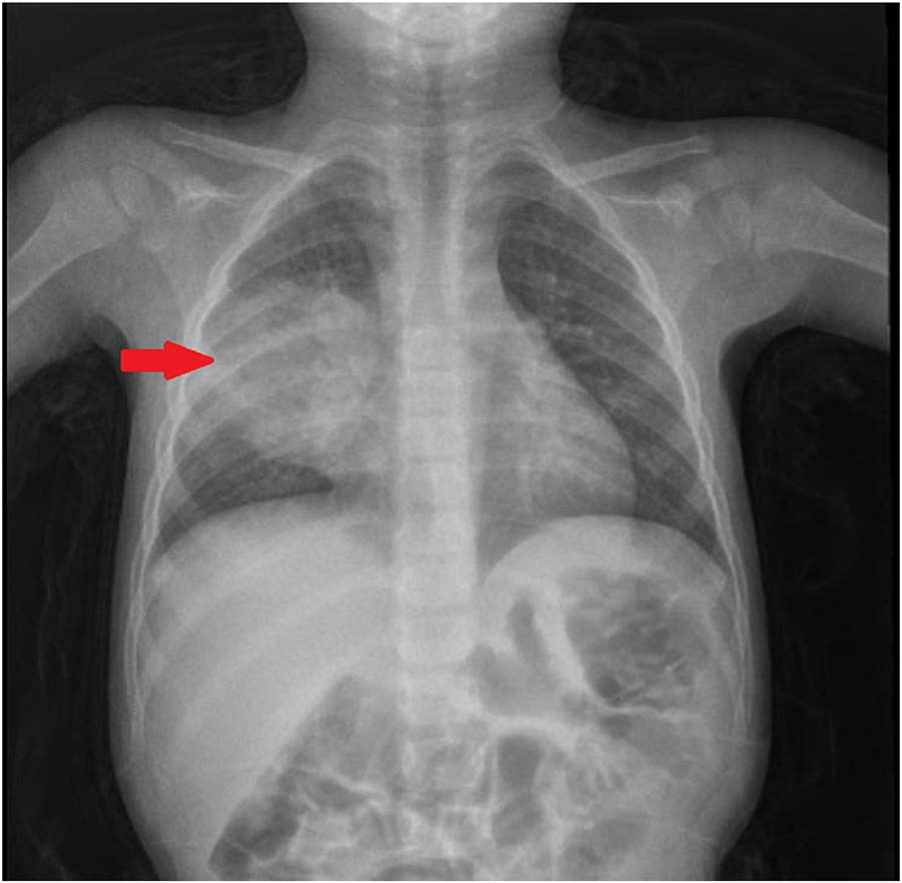
Chest radiograph demonstrating right upper and middle lobe consolidation.

**Figure 2:**
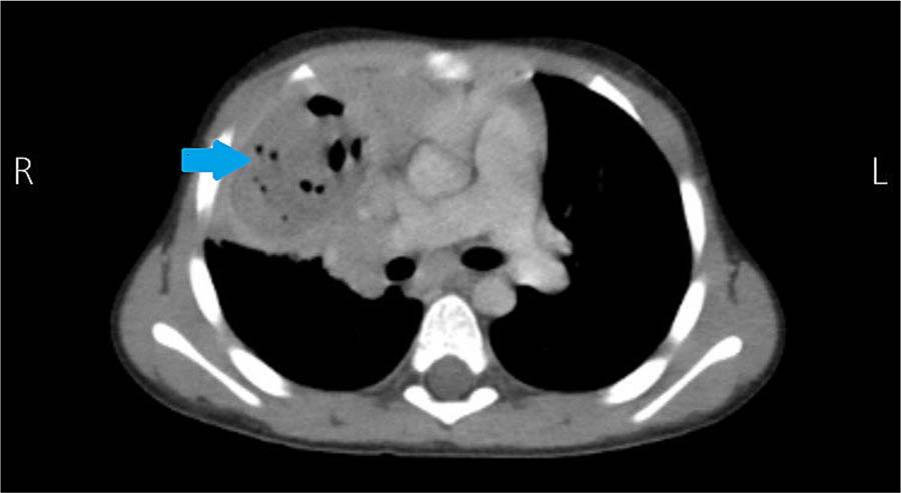
Contrast-enhanced CT showing right upper lobe cavitary lesion consistent with ruptured hydatid cyst.

Initial chest radiography showed consolidation involving the right upper and middle lobes (Fig. 1). Contrast-enhanced chest CT demonstrated a large (5 × 5 cm) heterogeneous cavitary lesion in the anterior segment of the right middle lobe with peripheral and internal enhancement, contrast leakage, surrounding atelectasis, parenchymal infiltration, and prominent mediastinal lymph nodes, raising suspicion for a ruptured pulmonary hydatid cyst (Fig. 2).

The patient was referred to our hospital on 3 January 2026 and underwent right-sided VATS-assisted excision of a pulmonary hydatid cyst on 4 January 2026.

The procedure was performed electively under general anesthesia with single-lumen endotracheal intubation (given the patient’s age). Bronchoscopy confirmed correct tube positioning, and no endobronchial lesions were identified. The patient was placed in the lateral decubitus position, and the surgical field was prepared and draped in a standard sterile fashion.

A 3-cm utility incision was made in the appropriate intercostal space, with stepwise opening of the chest wall layers, representing a VATS-assisted mini-thoracotomy approach. Intraoperatively, a hydatid cyst involving the right middle lobe was identified, adjacent to the pericardium, in the presence of pleural adhesions without effusion.

The cyst was injected with 40 ml of 3% hypertonic saline and left in situ for 5 minutes. Surrounding gauze packs soaked in hypertonic saline were applied to prevent spillage. The cyst was then opened, and the germinal layer was completely evacuated. Deroofing of the cyst was performed using LigaSure. The pleural cavity was irrigated with saline, and lung re-expansion revealed two bronchial openings, which were closed using interrupted 2/0 Vicryl sutures.

Capitonnage was performed using multiple 2/0 PDS sutures, achieving complete obliteration of the residual cavity. A 24 Fr chest tube was placed in the apicoposterior position. The lung was fully expanded, and the chest was closed in layers. The patient was extubated on table with no intraoperative complications and minimal blood loss.

Postoperatively, the patient demonstrated steady clinical and laboratory improvement. He tolerated oral intake, remained afebrile with stable vital signs, and had minimal chest tube output without air leak. Serial chest radiographs showed satisfactory lung re-expansion. The patient was discharged on 10 January 2026 in good general condition, with the chest tube connected to a drainage bag, and was prescribed albendazole along with supportive medications. Follow-up visits showed complete recovery with an excellent long-term prognosis.

## Discussion

Pulmonary cystic echinococcosis remains an important consideration in endemic regions, particularly in children, in whom lung involvement is relatively common and cysts may enlarge rapidly because of increased parenchymal compliance [6]. Infection follows ingestion of *Echinococcus* eggs, and pulmonary cysts often remain clinically silent until they reach a significant size or become complicated by bronchial communication, secondary infection, or rupture [7]. Ruptured pulmonary cysts pose a substantial risk due to airway contamination, pleural spillage, and potential allergic reactions, underscoring the importance of timely definitive management in symptomatic or complicated cases [6].

In the present case, a 4-year-old child presented with acute hemoptysis and productive cough, and chest CT demonstrated a large cavitary pulmonary lesion with surrounding atelectasis, raising suspicion for a ruptured hydatid cyst and prompting surgical referral. Rupture is a well-recognized complication in pediatric pulmonary HD and may produce characteristic imaging findings, such as detached membranes and air–fluid levels [8]. However, radiologic appearances can vary and may mimic lung abscess, congenital cystic lesions, or necrotizing pneumonia, making diagnosis challenging [9]. Consequently, in endemic settings, HD should remain high on the differential diagnosis for cystic or cavitary lung lesions in children.

Surgical intervention remains the cornerstone of treatment for pulmonary hydatid cysts, as it provides definitive source control and prevents ongoing contamination. The primary operative objectives include complete removal of the germinal layer and cyst contents, prevention of spillage, secure closure of bronchial openings, and appropriate management of the residual cavity [10]. In this case, a minimally invasive approach was successfully employed, with VATS-assisted mini-thoracotomy excision of the cyst, removal of the germinal membrane, closure of bronchial communications, and chest drainage, resulting in an uncomplicated postoperative course.

With increasing surgical experience, VATS-assisted mini-thoracotomy has emerged as a feasible alternative to thoracotomy in selected pediatric patients [11]. Pediatric series have demonstrated that VATS-assisted mini-thoracotomy offers comparable safety and efficacy while providing advantages in postoperative pain control, recovery time, and length of hospital stay, without compromising lung preservation [12]. Nevertheless, careful patient selection and meticulous adherence to anti-spillage principles remain essential. Intraoperative field isolation and the use of scolicidal agents, such as hypertonic saline, are commonly employed to minimize pleural contamination and recurrence risk [13].

Postoperative management plays a critical role in optimizing outcomes and preventing recurrence. Chest tube drainage facilitates lung re-expansion and early detection of air leaks, particularly in cases with bronchial communication [10]. Adjunct antiparasitic therapy with albendazole is widely recommended following surgical management, especially in ruptured cysts where the risk of intraoperative or preoperative spillage is increased [6, 14]. The World Health Organization supports the combined use of surgery and medical therapy in cystic echinococcosis to reduce recurrence and improve long-term outcomes [6].

In conclusion, this case illustrates that ruptured pulmonary hydatid cysts may present with acute hemoptysis even in very young children and that VATS-assisted mini-thoracotomy, when performed with meticulous anti-spillage technique, bronchial leak control, and appropriate adjunctive therapy, represents an effective lung-sparing treatment option with excellent short-term outcomes [11, 12].

Ruptured pulmonary hydatid cysts can present with massive hemoptysis even in very young children, posing diagnostic and therapeutic challenges. Early recognition and prompt surgical intervention, particularly using minimally invasive approaches such as VATS-assisted mini-thoracotomy, allow complete cyst removal, minimize complications, and preserve lung function. Adjunctive antiparasitic therapy with albendazole further reduces the risk of recurrence. Clinicians in endemic regions should maintain a high index of suspicion for pulmonary HD in children presenting with cystic or cavitary lung lesions, especially when complicated by hemoptysis, to ensure timely and effective management.

## Data Availability

Data are available from the corresponding author upon reasonable request.
